# Human Metapneumovirus, Peru

**DOI:** 10.3201/eid1202.051133

**Published:** 2006-02

**Authors:** Gregory C. Gray, Ana W. Capuano, Sharon F. Setterquist, Jose L. Sanchez, James S. Neville, James Olson, Mark G A Lebeck, Troy McCarthy, Yacine Abed, Guy Boivin

**Affiliations:** * University of Iowa College of Public Health, Iowa City, Iowa, USA;; † US Military HIV Research Program, Rockville, Maryland, USA;; ‡ Air Force Institute for Operational Health, Brooks City-Base, Texas, USA;; § US Navy Medical Research Center Detachment, Lima, Peru;; ¶ Centre Hospitalier Universitaire de Québec, Québec City, Québec, Canada

**Keywords:** Metapneumovirus, pneumovirinae, respiratory tract infections, epidemiology, genotyping, Latin America, Peru, Argentina

## Abstract

We retrospectively studied 420 pharyngeal swab specimens collected from Peruvian and Argentinean patients with influenzalike illness in 2002 and 2003 for evidence of human metapneumovirus (HMPV). Twelve specimens (2.3%) were positive by multiple assays. Six specimens yielded HMPV isolates. Four of the 6 isolates were of the uncommon B1 genotype.

Human metapneumovirus (HMPV) has been detected in patients with acute respiratory infection in North America, South America, Europe, Asia, the Middle East, Africa, and Oceania (*1**–**7*). Capitalizing on a preexisting US Department of Defense influenza surveillance system (*8*), we sought to detect and genotype HMPV in Latin American patients in whom influenzalike illness developed.

## The Study

Research was conducted on culture specimens collected from patients with influenzalike illness in Argentina and Peru under a US Department of Defense Global Emerging Infections System (GEIS) influenza surveillance program. Influenzalike illness is defined as fever (temperature >38°C) and cough or sore throat for <72 h. Under the GEIS influenza surveillance system (*8*), US and international sites collect posterior pharyngeal swabs for virus culture from patients who meet the influenzalike illness case definition.

Specimens were labeled with a unique specimen number and stored in cryovial boxes at –70°C until thawed for reverse transcription–polymerase chain reaction (RT-PCR) study. The specimens were linked by a unique laboratory number to an electronic database with patient's sex, age, collection date, city, and state.

After thawing to room temperature, the 420 swab specimens were screened with a 1-step RT-PCR procedure, with the F2 primer set. Briefly, RNA from each respiratory specimen was extracted with the QIAamp Viral RNA MiniKit (Qiagen, Valencia, CA, USA). The 1-step RT-PCR specimen screen was performed in a 100-μL reaction mix containing 11 μL RNA, 0.4 μmol/L forward primer, 0.2 μmol/L reverse primer, 0.163 mmol/L deoxynucleoside triphosphates, 100 U Moloney murine leukemia virus–reverse transcriptase, 10 U RNAse inhibitor, and 2.5 U DNA polymerase in 1× DNA polymerase buffer (*PfuTurbo*, Stratagene, La Jolla, CA, USA). Amplification conditions consisted of 1 h at 42°C; 5 min at 94°C; 34 cycles of 30 s at 94°C, 30 s at 52°C, and 1 min at 72°C; and a final extension at 72°C for 10 min. PCR products were analyzed by electrophoresis (BioRad, Hercules, CA, USA) in a 1.2% (wt/vol) agarose gel stained with ethidium bromide.

Screened specimens that gave bands within 200 bp of the expected 347-bp product were further tested with a 2-step RT-PCR with F1-, F2-, and N-gene primer sets. The 2-step RT-PCR was performed by using the RETROscript Kit (Ambion, Austin, TX, USA) with heat denaturation of RNA. PCR products were analyzed by gel electrophoresis. Specimens were designated RT-PCR–positive if the confirmatory N-gene primer set and at least 1 of the confirmatory F-gene primer sets yielded a band within 50 bp of the expected size (primers available from the corresponding author) (*9*).

Both 1- and 2-step RT-PCR procedures were adapted from previous reports (*9**–**12*). With every specimen batch, a known HMPV-positive and HMPV-negative sample was tested in parallel to validate the run.

RT-PCR–positive specimens were further studied with shell-vial cell culture for viable HMPV. A shell vial containing a near confluent monolayer of LLC-MK2 cells (Diagnostic Hybrids, Inc., Athens, OH, USA) was injected with 100 μL specimen and 900 μL HMPV growth media (1× minimum essential medium with L-glutamine and Earle salts, 0.1% bovine albumin, 1× HEPES, 0.001% porcine pancreatic trypsin, 0.4505 mol/L D-glucose, 10,000 U penicillin, 10 mg streptomycin, and 50 μg amphotericin), centrifuged for 1 h at 37°C and 2,800 rpm (1,500× *g*), followed by a 37°C incubation with 5% CO_2_. The cell monolayers were microscopically examined weekly for cytopathic effect (CPE) and contamination. Shell vials were incubated 3–4 weeks or until cell disruption occurred. Infected cell supernatant media were harvested each week upon cell media replacement. From an aliquot of the infected media, RNA was extracted and subsequent RT-PCR was performed by the HMPV F2-gene 1-step protocol.

Sequencing was performed on the RT-PCR–positive specimens by using G_univ_ primer set (available from the corresponding author), adapted to amplify an 800- to 1,000-bp region. Products were subsequently electrophoresed across a 1.0% agarose gel stained with ethidium bromide. RT-PCR–positive products were purified with QIAquick PCR Purification/Gel Extraction Kits (Qiagen). Strands of the amplicons were sequenced by automated sequencing with the G_univ_ primers. Big Dye Terminator Kit v3.1 (Applied BioSystems, Foster City, CA, USA) was used in sequencing reactions. Samples were run on a 3730xl DNA Analyzer (Applied BioSystems).

Alignments of partial amino acid sequences of the HMPV G protein were generated with the ClustalW software (National Center for Biotechnology Information, Bethesda, MD, USA). Prototypic sequences of different types (A and B) and subtypes (A1, A2, B1, and B2) from the Netherlands and Canada were included in the alignments. Phylogenetic analysis was performed by the neighbor-joining method by using MEGA 2 (University of Pittsburgh, Pittsburgh, PA, USA).

Specimen laboratory results were studied for demographic and temporal predictors of RT-PCR positivity by using standard categoric data techniques. Age group cut points were selected based on age quartiles. Exact binomial 95% confidence intervals (CIs) were calculated around prevalence statistics. Similarly, 95% CIs around odds ratios were calculated by using logistic regression. Analyses were performed by using SAS software version 9.1 (SAS Institute, Inc., Cary, NC, USA).

## Conclusions

We studied 420 posterior pharyngeal swab specimens collected from January 2002 to November 2003 (Table 1). Because of differences in clinic focus, the distribution of influenzalike illness differed by site; children made up higher proportions in each country (median age 11 years, range <1–89 years, Table 2). Overall, 51% of the 302 specimens for which patient's sex was known were from male patients. Most influenzalike illness specimens were obtained during the coldest months (July through September, data not shown).

**Table 1:** Prevalence and OR of RT-PCR positivity for HMPV by risk factor*

Risk factor	n	% RT-PCR–positive (95% CI)	OR (95% CI)
Age group (y)†			0.9 (0.8–0.99)
<7	151	6 (2.8–11)	7.2 (1–319.4)
7–20	152	1.3 (0.2–4.7)	1.5 (0.1–90.5)
>20	115	0.9 (0–4.8)	Reference
Unknown	2		
Sex			
Male	154	4.6 (1.9–9.1)	2.3 (0.5–14)
Female	148	2 (0.4–5.8)	Reference
Unknown	118		
Site			
Peru	388	2.8 (1.4–5)	Reference
Argentina	32	3.1 (0.1–16.2)	1.1 (0–8.1)
Season			
Autumn‡	106	6.6 (2.7–13.1)	4.3 (1.3–13.8)
Others	307	1.6 (0.5–3.8)	Reference
Unknown	7		

*OR, odds ratio, RT-PCR, reverse transcription–polymerase chain reaction; HMPV, human metapneumovirus; CI, confidence interval. †Age as a continuous variable. ‡Autumn in the Southern Hemisphere was considered to be from March 22 to June 21, per Centro de Divulgacao Cientifica e Cultural, São Paulo University, Brazil.

**Table 2:** Human metapneumovirus (HMPV)–positive samples by F- and N-gene primers

Case	Date collected	City, Country	Age (y)	F2 1-step (347 ± 200 bp)	F2 2-step (137 ± 50 bp)	N 2-step (212 ± 50 bp)	LLC-MK2 culture result
SA1131	6/02	Chanchamayo, Peru	9	+	+	+	No growth*
SA1066	6/02	Cuzco, Peru	2	+	+	+	No growth
SA1071	6/02	Cuzco, Peru	4	+	+	+	HMPV Peru2-2002
SA1226	10/02	Buenos Aires, Argentina	3	+	+	+	No growth†
SA1156	10/02	Cuzco, Peru	4	+	+	+	HMPV Peru1-2002
SA1385	4/03	Iquitos, Peru	5	+	+	+	No growth
SA3156	6/03	Iquitos, Peru	7	+	+	+	HMPV Peru3-2003
SA3157	6/03	Iquitos, Peru	4	+	+	+	HMPV Peru4-2003
SA3158	6/03	Iquitos, Peru	3	+	+	+	HMPV Peru5-2003
SA1532	8/03	Cuzco, Peru	0.75	+	+	+	No growth
SA1568	9/03	Cuzco, Peru	38	+	+	+	No growth
SA1606	10/03	Cuzco, Peru	0.75	+	+	+	HMPV Peru6-2003

*Bacteria contamination; specimen required filtering. †Mold contamination; specimen required filtering.

Twelve (2.9%) of 420 specimens were considered HMPV RT-PCR–positive (Table 2). All 12 positive specimens were cultured on LLC-MK2 cells. Six of the 12 specimens grew HMPV, and none of them showed evidence of viral CPE before 7 days. The nonviability of the 6 remaining positive specimens was likely due to the 4 freeze-thaw cycles that occurred before LLC-MK2 cell culturing or possibly the degradation of HMPV RNA within the specimens during transport and storage.

All 6 of the specimens that yielded an HMPV isolate in cell culture were successfully sequenced and were used to develop a phylogenetic tree (Figure) (*13*). Sequencing was not attempted until ≈2 years after specimen collection. This delay in sequencing and multiple freeze-thaw cycles may explain our inability to amplify and sequence G-gene product from the other 6 positive specimens. Sequence data were compared to previously sequenced HMPV isolates, showing a high prevalence of genotype B, with 4 isolates (Peru2-2002, Peru3-2003, Peru4-2003, and Peru5-2003) of the B1 subtype and 2 isolates (Peru1-2002 and Peru6-2003) of the B2 subtype. The high prevalence of genotype B isolates could be due to our methodologic approach and requires validation through other studies of similar Peruvian specimens.

**Figure:**
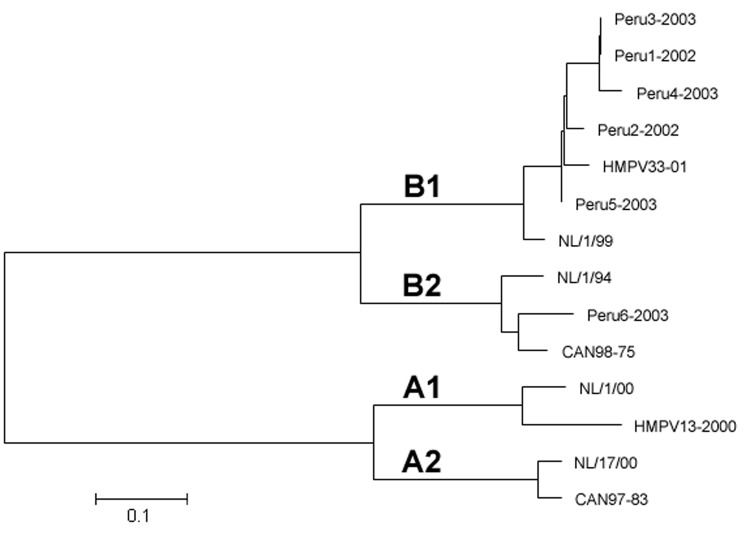
G-gene phylogenetic relationships of 6 human metapneumovirus (HMPV) isolates detected in South America during 2002 and 2003 compared to prototypic HMPV isolates from the Netherlands: NL/1/00, NL/17/00, NL/1/99, NL/1/94 (accession nos. AF371337, AY296021, AY525843, and AY296040, respectively) and from Canada: Can97-83, HMPV-13-00, CAN98-75, and HMPV-33-01 (accession nos. AY485253, AY485232, AY485245, and AY485242, respectively). Classification of genotypes was made according to previous reports (*13**,**14*).

Our data suggest that HMPV is circulating in Peru. Consistent with results of other studies, the prevalence of HMPV infection in this research was low among patients with influenzalike illness and more common among younger children (6% in children <7 years of age, Table 1) (*1*). In our study, HMPV was more often detected in male patients and from April to June.

Of the 12 HMPV RT-PCR–positive patients, 9 had clinical reports available for review. Three children from a small Peruvian Amazon village whose specimens were collected within 3 days of each other were infected with HMPV from the B1 subtype. Among these 3 children, the youngest (3 and 4 years of age) were the most debilitated and had the highest maximum oral temperature (39.8°C and 39.6°C). Among the remaining 6 HMPV-positive patients, 1 had pneumonia and 1 was hospitalized. These data show a higher likelihood (odds ratio 4.3, 95% CI 1.3–13.8) of detecting HMPV from patients with influenzalike illness during the Southern Hemisphere's autumn (March to June) (Table 1).

HMPV genotypes B1 and B2 were detected (Figure). Four of the 6 isolates belonged to genotype B1, which had been uncommon in Europe, Canada, and South Africa (*7**,**13**,**15*). These results represent some of first genotype data from HMPV isolates collected in Peru.
